# Efficiency of Single Phage Suspensions and Phage Cocktail in the Inactivation of *Escherichia coli* and *Salmonella* Typhimurium: An *In Vitro* Preliminary Study

**DOI:** 10.3390/microorganisms7040094

**Published:** 2019-03-31

**Authors:** Pedro Costa, Carla Pereira, Ana T. P. C. Gomes, Adelaide Almeida

**Affiliations:** Department of Biology and CESAM, University of Aveiro, Campus Universitário de Santiago, 3810-193 Aveiro, Portugal; pedrommrscosta@ua.pt (P.C.), csgp@ua.pt (C.P.), ana.peixoto@ua.pt (A.T.P.C.G.)

**Keywords:** bacterial-phage inactivation, phage cocktail, *Escherichia coli*, *S.* Typhimurium

## Abstract

Enterobacteriaceae *Escherichia coli* and *Salmonella enterica* serovar Typhimurium strains are among the main pathogens responsible for moderate and serious infections at hospital and community environments, in part because they frequently present resistance to antibiotics. As the treatment of Enterobacteriaceae infections is empiric, using the same antibiotics to treat *E. coli* and *Salmonella* infections, the same concept can be applied with phages. The use of different phages combined in cocktails, frequently used to circumvent the development of phage-resistant mutants, also allows for the treatment of multiple pathogens, broadening the phages’ action spectrum. As such, the aim of this study was to evaluate the efficiency of a cocktail of two phages (ELY-1, produced on *E. coli* and phSE-5, produced on *S.* Typhimurium) to control *E. coli* and *S.* Typhimurium. Phages ELY-1 and phSE-5 were effective against *E. coli* (maximum reductions of 4.5 and 3.8 log CFU/mL, respectively), *S.* Typhimurium (maximum reductions of 2.2 and 2.6 log CFU/mL, respectively), and the mixture of both bacteria (maximum reductions of 2.2 and 2.0 log CFU/mL, respectively). The cocktail ELY-1/phSE-5 was more effective against *S.* Typhimurium and the mixture of both bacteria (maximum reduction of 3.2 log CFU/mL for both) than the single phage suspensions and as effective against *E. coli* as its specific phage ELY-1 (maximum reductions of 4.5 log CFU/mL). The use of both the phage cocktails, as well as the single-phage suspensions, however, did not prevent the occurrence of phage-resistant mutants. Overall, the results indicate that the application of the phages in the form of a cocktail show their potential to be used presumptively, that is, prior to the identification of the pathogens, paving its use to control *E. coli* or *S.* Typhimurium.

## 1. Introduction

*Escherichia coli* and *S.* Typhimurium are the major bacterial pathogens associated with many cases of serious infections. *E. coli* is a non-pathogenic commensal bacterium categorized by its versatility and assortment once it is capable of colonizing human and other animal gastrointestinal systems [1,2,3,4]. However, new virulent strains appear due to the evolution of some strains, which are responsible for varied diseases, such as urinary tract infections (UTI), meningitis intestinal, septicemia, diarrhea, and pneumoniae [5,6,7]. There are several *E. coli* strains that promote enteric diseases, such as adherent invasive *E. coli* (AIEC), diffusely adhering *E. coli* (DAEC), enterotoxigenic *E. coli* (ETEC), enteropathogenic *E. coli* (EPEC), Shiga-toxin-producing enteroaggregative *E. coli* (STEAEC), enteroinvasive *E. coli* (EIEC), enterohaemorrhagic *E. coli* (EHEC) and enteroaggregative *E. coli* (EAEC) [5,8,9,10]. Neonatal meningitis-associated *E. coli*, sepsis-causing *E. coli*, and uropathogenic *E. coli* (UPEC) are the extraintestinal pathogenic *E. coli* strains [11]. 

*Salmonella* has been known as one of the important foodborne pathogens and a major public health burden worldwide. *Salmonella enterica* serovar Enteritidis and serovar Typhimurium are the main photogenic agents of a high number of enteric infections in the world, transmitted by cross-contamination of ready-to-eat products and raw foods [12]. Gastroenteritis is mainly caused by *Salmonella* and the symptoms include diarrhea, abdominal cramps, fever, and nausea [3,4,13]. Other clinical manifestations can include urinary tract infections, bacteraemia and septicaemia [14].

The rapid emergence of antimicrobial multidrug-resistant bacteria occurring worldwide has been attributed to the overuse of antibiotics. Currently, the increased occurrence and prevalence of antibiotic resistance in *E. coli* and *Salmonella* are a particular concern [15,16]. One of the most problematic areas of drug resistance is the resistance acquired by fluoroquinolones and third generation cephalosporin by Enterobacteriaceae, which include strains of *E. coli* and *Salmonella*, according to the World Health Organization (WHO) [17,18].

For the reduction of the development and dissemination of microbial resistance, alternative strategies must be developed [19,20]. A promising alternative for the treatment of infections is the use of phages as antibacterial agents, mainly those caused by multidrug-resistant bacteria [21,22,23,24,25]. The major advantages for the application of phage therapy over antibiotics are the specific targeting, since phages are usually highly specific to a single species or even strain of bacteria, which can cause less damages to microbiota; a limited impact since phages are self-replicating and self-limiting, phages replicate exponentially like bacteria, and decline when bacteria number decreases [14,26,27,28,29,30,31]. Some studies have demonstrated that phages can be used to successfully prevent or control *E. coli* [20,32,33,34,35,36,37,38] and *S.* Typhimurium [14,39,40,41,42,43,44]. The major concern of this alternative therapeutic approach is regarding the use of phages in the treatment of infectious diseases due to the regrowth of bacteria after treatment, a consequence of the emergence of phage-resistant mutants [45]. The development of phage-resistant bacteria has been attributed to genetic changes [46], however, it has been reported that bacterial populations may maintain their viability in the presence of phages due to phenotypic resistance, remaining genetically sensitive to them [47,48]. It has been stated that phage-resistance development can be overcome by the combined use of two or more phages through phage cocktails [14,20,49,50].

Phage cocktails allow the treatment of multiple pathogens and potentially provide a mean to circumvent resistance to a present single phage [51,52,53]. Consequently, the high phages specificity, that is often considered a disadvantage of phage therapy, namely when pathogenic bacteria are not known, may be avoided by using phage cocktails, since they broaden the spectrum of action [49,54]. In fact, numerous studies have demonstrated the great potential of the used of phage cocktails in the inactivation of infections [14,20,32,35,41,42]. Major cocktail formulations against enteric bacteria (Intestiphage and Pyophage), marketed in Georgia and Russia, were originally developed by D’Herelle in the Pasteur Institute in the 1930s [55] and the ColiProteus cocktail is produced by the Russian company Microgen [56]. These cocktails are subject to standard testing against relevant pathogens every 6 months and their composition is adjusted to meet current needs [55]. Moreover, to our knowledge, only one study evaluated the therapeutic potential of phages to control a mixture of bacteria [57]. These authors evaluated the potential therapeutic effect of three phages isolated against *Enterobacter cloacae* el140, *Klebsiella pneumoniae* kp235, and *E. coli* ec31 using *Galleria mellonella* as an animal model [57]. The results indicated that all three phages had the potential to infect the host bacterial strains and multiple doses of the phage cocktail were necessary to recover the larvae from the mixed bacterial infection. 

The goal of this study was the efficiency evaluation of a cocktail of two phages (ELY-1, produced on *E. coli* and phSE-5, produced on *S.* Typhimurium) to control infections caused by *E. coli* or *S.* Typhimurium. For this, the single phage suspensions (ELY-1 and phSE-5) and the phage cocktail of these two phages (ELY-1/phSE-5) were tested to inactivate *E. coli*, *S.* Typhimurium and the mixture of *E. coli* and *S.* Typhimurium. The rate of emergence of phage resistant mutants and the fitness of these mutants were also determined for *E. coli*, *S.* Typhimurium and the mixture of the two bacteria after treatment with phages ELY-1, phSE-5 and the phage cocktail ELY-1/phSE-5.

## 2. Materials and Methods

### 2.1. Bacterial Strains and Growth Conditions

The bacterial strains *E. coli* [58] and *S.* Typhimurium (ATCC 13311) were used in this study as phage hosts. *E. coli* Scc 09, 34, 35, 36, 37, 38, 40, 41, 43, 45, 47, 48, 50, 51, 52, 53, 55, 56, 58, 69, 77, 78, and 91 were isolated from an urban wastewater treatment plant [59]. Five *S.* Enteriditis strains were isolated from food samples and gently provided by Controlvet Laboratory. The other bacterial strains used in this study were isolated in previous works from water samples collected in Ria de Aveiro [58,60].

All bacteria were grown in Tryptic Soy Broth (TSB, Liofilchem, Italy). Fresh plate bacterial cultures were maintained in Tryptic Soy Agar medium (TSA; Liofilchem, Italy) at 4 °C. Before each assay, one isolated colony was aseptically transferred to 30 mL of TSB and grown overnight at 25 °C at 120 rpm stirring. An aliquot of this culture (100 µL) was transferred to 10 mL of fresh TSB medium and grown overnight at 25 °C to reach an optical density (O.D. 600) of 0.8, corresponding to about 10^9^ cells/mL.

### 2.2. Phage Preparation

The phages ELY-1 and phSE-5 were isolated in previous works from water samples collected from the Corte das Freiras aquaculture [61] and from the sewage network of Aveiro (station EEIS9 of SIMRIA Multi Sanitation System of Ria de Aveiro) [14], respectively. The phage ELY-1 was identified as double-stranded DNA phage of the order Caudovirales, family *Myoviridae*, a T4-like phage with 95% of homology with the Enterobacteriaceae phage vB_EcoMVR7 (accession number HM563683) [61]. Phage phSE-5 was identified as double-stranded DNA phage of the order Caudovirales, family *Siphoviridae*, with 94% homology with the *Siphoviridae* phages, TLS (accession numberAY308796.1), and *Salmonella* phage FSL SP-126 (accession numberKC139513.1) [14]. 

Phages ELY-1 and phSE-5 were prepared using *E. coli* and *S.* Typhimurium as the host, respectively. Phage suspensions were prepared from the phage stock prepared previously in SM buffer (0.1 M NaCl (Sigma-Aldrich, St. Louis, MO, USA), 8 mM MgSO_4_ (Sigma), 20 mM Tris-HCl (Sigma), 2% (w/v) gelatin, pH 7.5). Three hundred microliters of the phage stock were added to 30 mL of *E. coli* or *S.* Typhimurium in the exponential growth phase. The suspension was grown overnight and incubated at 25 ˚C at 50 rpm. The lysates were incubated with chloroform (final volume of 1%) for 1 h at 120 rpm. After incubation, the lysate was centrifuged at 13,000 rpm for 10 min at 4 ˚C, to remove intact bacteria or bacterial debris. Phage suspension was stored at 4 °C and the titre was determined by the double-layer agar method [62]. Successive dilutions of the phage suspension were performed in PBS and 500 μL of each dilution, together with 200 μL of fresh bacterial culture, and were mixed with 5 mL of TSB 0.6% top agar layer (30 g/L TSB (Liofilchem), 6 g/L agar (Liofilchem), 0.05 g/L CaCl_2_ (Sigma), 0.12 g/L MgSO_4_ (Sigma), pH 7.4) and placed over a TSA plate. The plates were incubated at 25 °C for 8–12 h. After incubation, the number of plaques was counted and the results expressed as plaque-forming units (PFU)/mL.

### 2.3. Phage Host Range: Spot Test and Efficiency of Plating (EOP)

The phage host range was determine using single suspensions (ELY-1 and phSE-5) and the bacterial strains listed in Table 1. The phage host range was determined by a spot test according to Pereira et al. (2016) [14]. Briefly, three milliliters of TSB 0.6% agar (Merck, Darmstadt, Germany), previously inoculated with 300 μL of bacterial culture (see Table 1), were overlaid on solid TSA and spotted with 10 μL of the phage suspension. The plates were incubated at 25 °C and examined for lysis plaques after 8–12 h. Bacterial sensitivity of the phage was established by a lysis cleared zone at the spot. According to the clarity of the spot, bacteria were differentiated into two categories, clear lysis zone (+) and not lysis zone (−). 

The EOP was determined for bacteria where a clear lysis zone occurred (positive spot test), using the double-layer agar method. The EOP was calculated as the ratio between the PFU average on the target bacteria and the PFU average on the host bacteria. EOP values are presented in the manuscript as the mean of three measurements followed by their standard deviation. The value obtained with the host strain was considered as EOP = 1.

### 2.4. Killing Curves

Bacterial inactivation was determined using single phage suspensions (ELY-1 and phSE-5) and a phage cocktail (ELY-1/phSE-5, the two phages were mixed together with each phage at the same concentration) using the bacterium *E. coli* or *S.* Typhimurium or a mixture of both bacteria with the same concentration, at a MOI of 100. Exponential bacterial cultures of *E. coli*, *S.* Typhimurium, or the mixture of the two bacteria were adjusted to a 0.8 O.D. at 600 nm (corresponding to a cell density of 10^9^ CFU/mL). In order to obtain a MOI of 100, the exponential cultures of bacteria (final concentration of 10^6^ CFU/mL) and phage suspension (final concentration of 10^8^ PFU/mL) were inoculated in sterilized glass Erlenmeyer flasks with 30 mL of TSB medium and incubated at 25 °C without agitation (B+P). For each assay, two control samples were included, as follows: The bacterial control (BC) and the phage control (PC). The bacterial control was not inoculated with phages and the phage controls were inoculated with phages but not with bacteria. Controls and test samples were incubated in exactly the same conditions. Aliquots of the test samples and of the bacterial and phage controls were collected at time 0 and after 2, 4, 6, 8, 10, and 12 h of incubation. The phage titre was determined in duplicate through the double-agar layer method after an incubation period of 12 h at 25 °C. Bacterial concentration was determined in duplicate in TSA medium after 48 h at 25 °C. Sensitive and phage resistant colonies were picked and purified by successive sub-culturing in TSA agar in order to remove attached phage particles and were used in further experiments (as described in Section 2.6, Fitness of Phage Resistant Mutants). Three independent experiments were performed for each condition.

### 2.5. Determination of the Frequency of Emergence of Phage Resistant Mutants

The development of resistant mutants of *E. coli* and *S.* Typhimurium, in pure and mixed cultures of phages ELY-1, phSE-5, and phage cocktail ELY-1/phSE-5, were evaluated according to Pereira et al. (2016) [14]. To determinate the frequency of phage-resistant bacteria, ten isolated colonies from a plate with sensitive bacteria were selected and inoculated into ten tubes with 5 mL of TSB, grown at 25 °C for 12 h at 120 rpm stirring. Aliquots of 100 µL from the 10^−1^ to 10^−3^ dilutions of the bacterial culture and of the phage, from a stock solution at 10^9^ PFU/mL, were inoculated in tubes with TSB 0.6% agar, plated on TSA plates and incubated at 25 °C for 24 h. Simultaneously, 100 µL aliquots of the 10^−5^ to 10^−7^ dilutions of the bacterial culture were plated by incorporation on TSA plates without phages and incubated at 25 °C for 24 h. The averaged colony number of mutants (obtained from the ten isolated colonies) in 1 mL of culture (prepared from the culture with phages) was divided by the averaged colony number of the control (prepared from the culture without phages) [63]. Three independent assays were performed.

### 2.6. Fitness of Phage Resistant Mutants

The growth of resistant and sensitive bacterial populations was quantified in the presence and absence of the phages ELY-1, phSE-5, and the phage cocktail ELY-1/phSE-5, in order to evaluate the toll bacteria suffers (“the fitness”) to develop resistance to the phages. Exponential host bacterial cultures of *E. coli*, *S.* Typhimurium, and the mixture with the two bacteria (without phage contact) and mutants resistant to the phages ELY-1, phSE-5, and the phage cocktail ELY-1/phSE-5 were adjusted to 0.8 O.D. at 600 nm (corresponding to a cell density of 10^9^ CFU/mL). The fitness of each bacterial population was evaluated by determining the bacterial concentration using the colony-counting method and by determining the bacterial growth curve using the optical density (OD). 

Pure cultures of *E. coli* or *S.* Typhimurium or the mixture of the two bacteria resistant to phage ELY-1 were added to 6 samples to obtain a final concentration of 10^6^ CFU/mL. Three of these samples were inoculated with a phage (resistant bacteria with phage ELY-1) to obtain a final concentration of 10^8^ PFU/mL. To the remaining infected samples, no phages were added (resistant bacteria without phages). The same protocol was followed for the mutants resistant to phage phSE-5 and to the cocktail ELY1/phSE-5. The same procedure was done for *E. coli* or *S.* Typhimurium or the mixture with the two bacteria using sensitive bacteria. Samples were incubated at 25 °C and bacterial concentration was determined by the spread method in duplicate in TSA medium at time 0 and after 6 and 12 h. Three independent experiments were performed for each condition.

In parallel, OD600 nm was measured at 0, 2, 4, 6, 8, 10, and 12 h after inoculation, using a spectrophotometer (Halo DB-20, Dynamica Scientific, Newport Pagnell, UK). Three independent experiments were performed for each condition.

### 2.7. Statistical Analysis

The statistical analysis of data was performed using GraphPad Prism 7.04 software. Normal distribution was checked by a Kolmogorov–Smirnov test and the homogeneity of variance was assessed by Levene’s test. Significance was accepted at *p* < 0.05. Tukey’s multiple comparison test was used for a pairwise comparison of the means. The significance of bacterial and viral concentrations between treatments, and along the experiments, was tested using two-way ANOVA and the Bonferroni post-hoc test (Section 3.2, Section 3.3 and Section 3.4). For different treatments, the significance of differences was evaluated by comparing the result obtained in the test samples with the results obtained for the correspondent control samples, for the different times. One-way ANOVA was used to examine the differences between the frequency of bacteria spontaneous phage-resistant mutants (Section 3.5). Two-way ANOVA was used to examine differences between the concentration of resistant bacteria and sensitive bacteria in the presence/absence of the phage after 6 and 12 h of incubation (Section 3.6). Two-way ANOVA with repeated measures was used to analyze the statistical differences between growth curves of the sensitive and resistant bacteria in the presence and absence of the phages during the sampling time (Section 3.6).

## 3. Results

### 3.1. Phage Host Range: Spot Test and Efficiency of Plating (EOP)

The results of spot test indicated that phage phSE-5 was capable of forming cleared zones on 25 of the 50 strains and the phage ELY-1 formed cleared zones on 9 of the 50 strains tested (Table 1). However, EOP results showed that phages ELY-1 and phSE-5, besides their host, formed phage lysis plates in only 4 strains of the 50 strains tested. Phage ELY-1 infected *S.* Typhimurium ATCC13311, *E. coli* ATCC 25922, *E. coli* Scc 34, and *E. coli* Scc 69 with an efficacy of 5.7 × 10^−4^ ± 2.5 × 10^−5^, 22.7 ± 1.02, 5.5 × 10^−5^ ± 5.0 × 10^−6^ and 2.8 × 10^−1^ ± 8.7 × 10^−2^. Phage phSE-5 infected bioluminescent *E. coli*, *E. coli* AE11, *E. coli* Scc 34 and *E. coli* Scc 69 with an efficacy of 2.2 × 10^−1^ ± 1.5 × 10^−2^, 1.7 × 10^−8^ ± 5.6 × 10^−9^, 1.8 × 10^−5^ ± 1.5 × 10^−6^ and 2.5 × 10^−8^ ± 4.8 × 10^−9^.

### 3.2. Effect of phage ELY-1, phSE-5, and cocktail ELY-1/phSE-5 on the inactivation of E. coli

**Figure 1:**
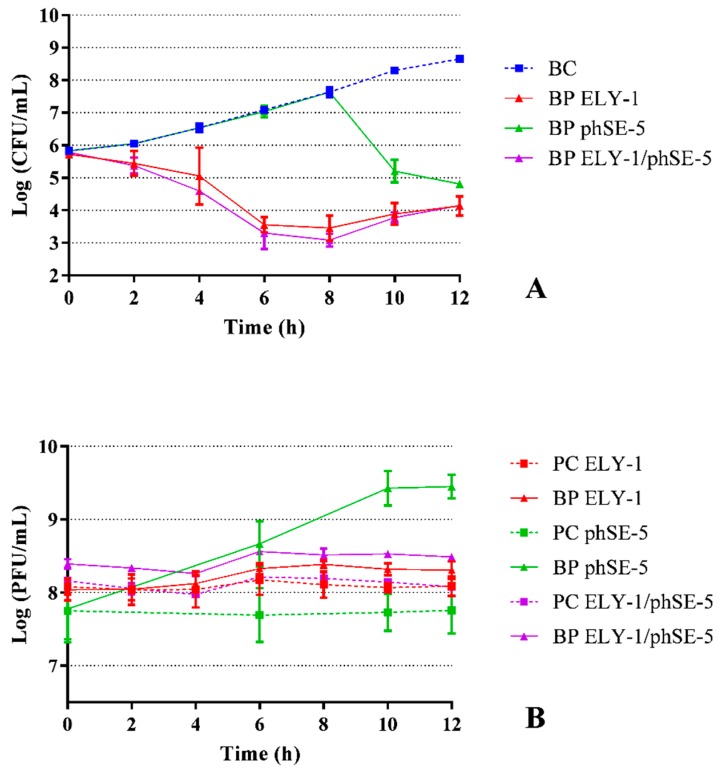
Inactivation of *E. coli* by two phages (ELY-1 and phSE-5) and the phage cocktail (ELY-1/phSE-5) at a MOI of 100 during 12 h. (**A**) Bacterial concentration: BC, bacteria control; BP, bacteria plus phage. (**B**) Phage concentration: PC, phage control; BP, bacteria plus phage. Values represent the mean of three independent assays; error bars represent the standard deviation.

The maximum inactivation with phage ELY-1, phSE-5, and cocktail ELY-1/phSE-5 was 4.5, 3.8, and 4.5 log CFU/mL, respectively, achieved after 12 h of incubation (ANOVA, *p* < 0.05, Figure 1A), when compared with those of the bacterial control (BC). However, after 6 h of incubation, the rate of bacterial inactivation with phage ELY-1 and the phage cocktail ELY-1/phSE-5 (3.5 and 3.8 log CFU/mL, respectively) was already high (ANOVA, *p* < 0.05) and almost fourfold higher than that obtained with the phage phSE-5 (reduction of 0.05 log CFU/mL, Figure 1A). Bacterial density in the BC increased by 2.8 log CFU/mL (ANOVA, *p* < 0.05, Figure 1A) during 12 h of incubation.

The phage controls (PC) remained constant during the 12 h of the experiments (ANOVA, *p* > 0.05, Figure 1B). For phage ELY-1 and the phage cocktail, the survival factor increased up to 0.5 log PFU/mL (ANOVA, *p* < 0.05) after 12 h of incubation. When the phage phSE-5 was incubated in the presence of *E. coli*, a significant increase (1.7 log PFU/mL, ANOVA, *p* < 0.05) was observed after 10 h of incubation.

### 3.3. Effect of Phage ELY-1, phSE-5 and Cocktail ELY-1/phSE-5 on the Inactivation of S. Typhimurium

**Figure 2:**
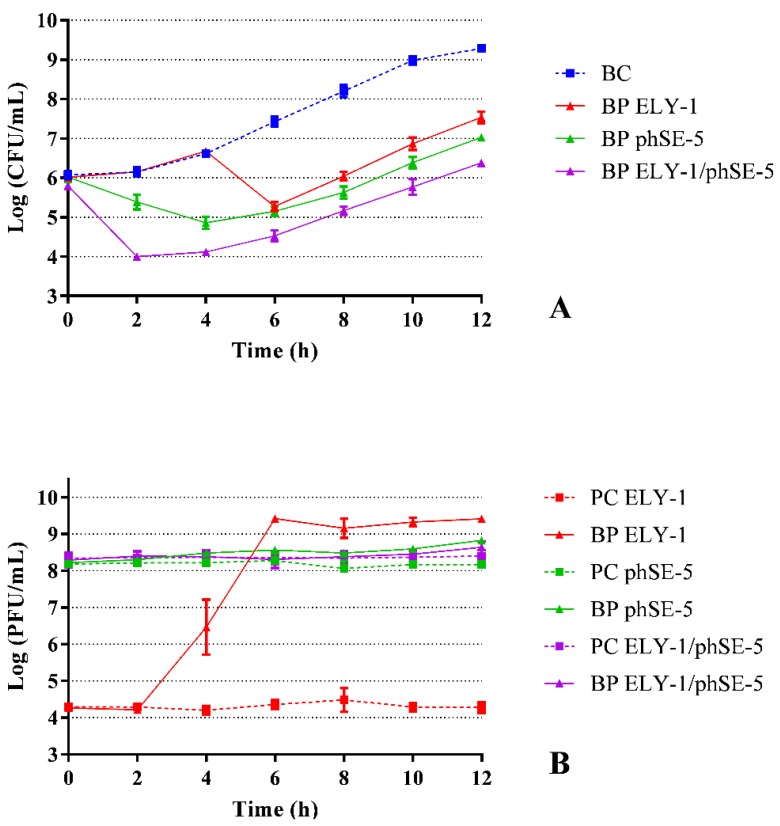
Inactivation of *S*. Typhimurium by two phages (ELY-1 and phSE-5) and the phage cocktail (ELY-1/phSE-5) at a MOI of 100 during 12 h. (**A**) Bacterial concentration: BC, bacteria control; BP, bacteria plus phage. (**B**) Phage concentration: PC, phage control; BP, bacteria plus phage. Values represent the mean of three independent assays; error bars represent the standard deviation.

The maximum inactivation of *S.* Typhimurium with phages ELY-1, phSE-5, the and cocktail ELY-1/phSE-5 was 2.2, 2.6, and 3.2 log CFU/mL (ANOVA, *p* < 0.05) achieved after 8, 10, and 10 h of incubation, respectively, when compared with those of the bacterial control (BC) (Figure 2A). After 2 and 4 h of treatment, the rate of bacterial inactivation with phage phSE-5 (0.8 and 1.8 CFU/mL, respectively) was significantly higher (ANOVA, *p* < 0.05) than the one obtained with phage ELY-1 (bacterial concentration similar to the bacterial control). However, after 6 h of treatment, the bacterial inactivation of phage ELY-1 was similar (2.2 log CFU/mL, ANOVA, *p* > 0.05) to that obtained with phage phSE-5 (reduction of 2.3 log CFU/mL) (Figure 2A). In general, the rate of inactivation of the phage cocktail was significantly higher than the one obtained with phages ELY-1 and phSE-5. After 12 h, the rate of inactivation was still considerably high (1.8, 2.3, and 3.0 log CFU /mL for phage ELY-1, phSE-5 and cocktail phSE-5/ELY-1, ANOVA, *p* < 0.05) (Figure 2A). Bacterial density in the BC increased by 3.2 log CFU/mL (ANOVA, *p* < 0.05, Figure 2A) during 12 h of incubation. 

The phage controls (PC) remained constant during the 12 h of the assay (ANOVA, *p* > 0.05, Figure 2B). In the case of the phages incubated in the presence of *S.* Typhimurium, a significant increase (ANOVA, *p* < 0.05) of 0.4 and 2.3 log PFU/mL was observed for ELY-1 and phSE-5, respectively, after 8 and 4 h of incubation (Figure 2B). In the case of the phage cocktail incubated in the presence of the *S.* Typhimurium (BP), no significant difference was observed when compared with the phage control (ANOVA, *p* > 0.05, Figure 2B).

### 3.4. Effect of Phages ELY-1, phSE-5 and Cocktail ELY-1/phSE-5 on the Inactivation of the Mixture of E. coli/S. Typhimurium

**Figure 3:**
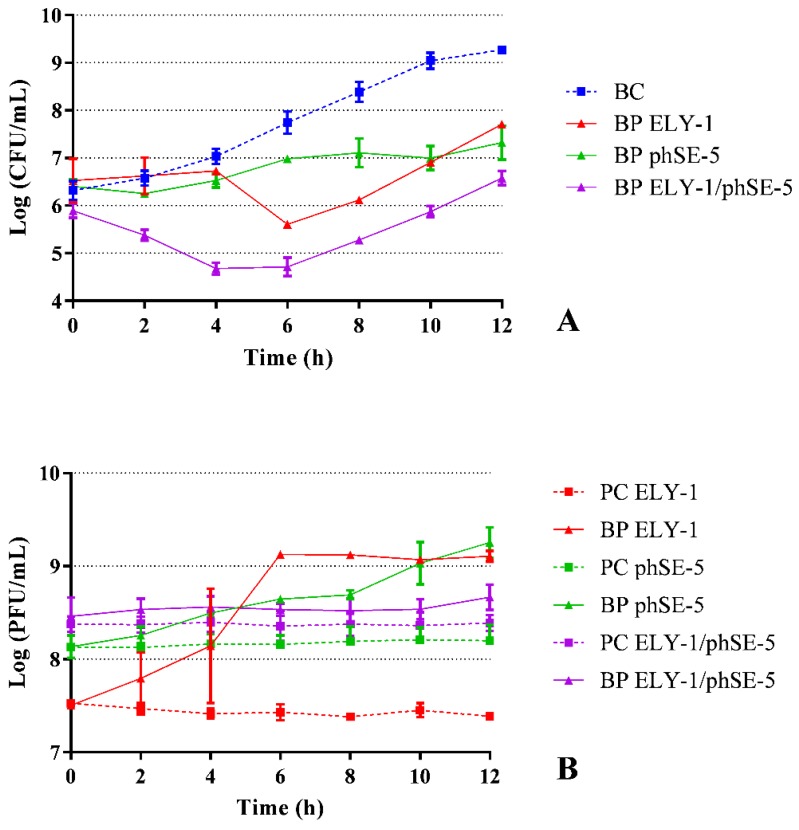
Inactivation of the mixture culture of *E. coli* and *S.* Typhimurium by two phages (ELY-1 and phSE-5) and the phage cocktail (ELY-1/phSE-5) at a MOI of 100 during 12 h. (**A**) Bacterial concentration: BC, bacteria control; BP, bacteria plus phage. (**B**) Phage concentration: PC, phage control; BP, bacteria plus phage. Values represent the mean of three independent assays; error bars represent the standard deviation.

The maximum inactivation of the mixture of *E. coli*/*S.* Typhimurium with the phages ELY-1, phSE-5, and cocktail phSE-5/ELY-1 was 2.2, 2.0, and 3.2 log CFU/mL (ANOVA, *p* < 0.05) achieved after 8, 10, and 10 h, respectively, when compared with those of the bacterial control (BC) (Figure 3A). During treatment, the rate of inactivation of the phage cocktail was significantly higher than that obtained with phages ELY-1 and phSE-5. The bacterial inactivation of the phage cocktail started after 2 h and the rate of inactivation (1.2 log) was significantly higher (ANOVA, *p* < 0.05) than those of phages ELY-1 and phSE-5 (bacterial concentration similar to bacterial control). However, after 6 h of treatment, the rate of inactivation of phage ELY-1 was significantly higher (2.1 log CFU/mL, ANOVA, *p* < 0.05) than that obtained with phage phSE-5 (0.8 log CFU/mL) (Figure 3A) and significantly lower than that obtained with the phage cocktail (3.0 log CFU/mL). After 12 h of incubation, the bacterial inactivation was 2.7 log CFU/mL with the phage cocktail, which was statistically different (ANOVA, *p* < 0.05) from the values obtained in the treatment with phages ELY-1 and phSE-5 (1.6 and 2.0 log CFU/mL, respectively). Bacterial density in the BC increased by 3.0 log CFU/mL (ANOVA, *p* < 0.05, Figure 3A) during 12 h of incubation. 

In the case of phages phSE-5, ELY-1, and the phage cocktail controls (PC) no decrease of the phage survival (ANOVA, *p* > 0.05) was observed during the 12 h of the experiments (Figure 3B). When the phages ELY-1 and phSE-5 were incubated in the presence of the mixture of bacteria, a significant increase (ANOVA, *p* < 0.05) of 1.7 and 0.5 PFU/mL was observed, respectively, after 6 h of incubation (Figure 3B). No significant differences were observed between the phage concentration and the phage control during the experiment with the phage cocktail (ANOVA, *p* > 0.05).

### 3.5. Determination of the Emergence Rate of Bacterial Mutants

*E. coli*, *S.* Typhimurium, and the mixture with the two bacteria showed different rates of resistant mutants when subjected to phages ELY-1, phSE-5, and the phage cocktail ELY-1/phSE-5 (Table 2). 

The development of *E. coli* mutants resistant to phage ELY-1 (5.06 × 10^−5^) was significantly lower (ANOVA, *p* < 0.05) than that obtained with phage phSE-5 (1.29 × 10^−3^) and the phage cocktail ELY-1/phSE-5 (1.22 × 10^−3^). However, when *E. coli* was inoculated with the phage cocktail, the emergence rate of resistance was similar (ANOVA, *p* < 0.05) to that obtained with phage phSE-5.

The frequency of *S.* Typhimurium resistant mutants in the presence of the phage ELY-1 (5.11 × 10^−^^5^) was significantly lower (ANOVA, *p* < 0.05) to that obtained with phage phSE-5 (2.80 × 10^−4^) and the phage cocktail ELY-1/phSE-5 (2.75 × 10^−4^). The frequency of *S.* Typhimurium resistant mutants in the presence of the phage phSE-5 and the phage cocktail ELY-1/phSE-5 was similar (ANOVA, *p* > 0.05).

The development of mutants in the mixture of bacteria when the phage cocktail ELY-1/phSE-5 was present (2.75 × 10^−4^) was significantly lower (ANOVA, *p* < 0.05) than that obtained with phages ELY-1 (4.66 × 10^−4^) and phSE-5 (5.09 × 10^−4^). 

### 3.6. Fitness of Phage Resistant Mutants

**Figure 4:**
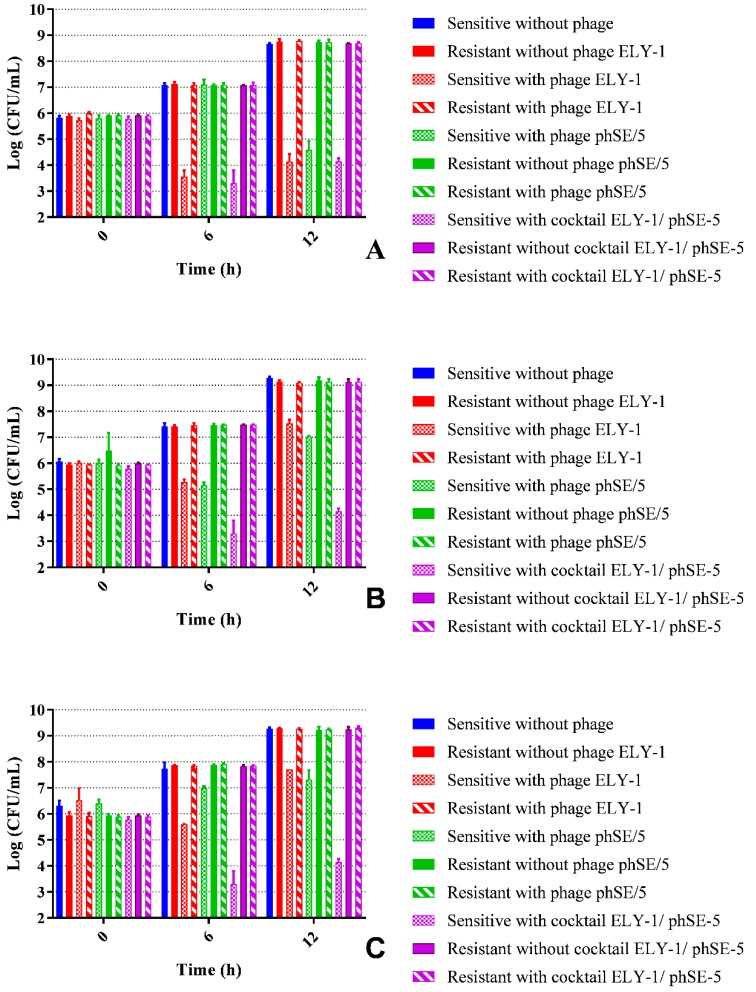
*Invitro E. coli* (**A**), *S.* Typhimurium (**B**), and mixture of *E. coli* and *S.* Typhimurium (**C**) concentration of resistant mutants versus their sensitive bacteria in the presence or absence of phage ELY-1, phSE-5, the and phage cocktail ELY-1/phSE-5, after 6 and 12 h.

In all experiments, in the presence of phages ELY-1, phSE-5, and the cocktail, differences were observed (Figure 4A–C, ANOVA, *p* < 0.05) between the growth profiles of sensitive bacteria and resistant bacteria. Higher concentrations of resistant bacteria to the phages or to the phage cocktail than sensitive bacteria at 6 and 12 h were observed. In the absence of phages, no differences (Figure 4A–C, ANOVA, *p* > 0.05) were found between the concentration of resistant bacteria, using the single phage suspensions or the cocktail, and the concentration of sensitive bacteria.

**Figure 5:**
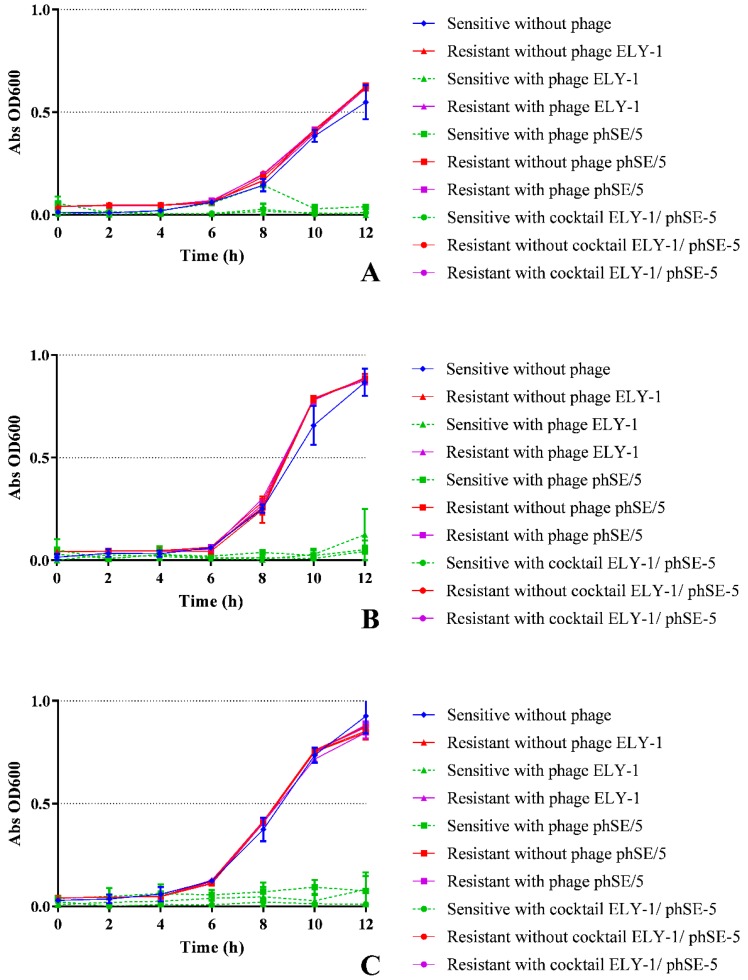
*In vitroE. coli* (**A**), *S.* Typhimurium (**B**), and mixture of *E. coli* and *S.* Typhimurium (**C**) density (optical density readings at 600 nm) of resistant mutants versus their sensitive bacteria in the presence or absence of phage ELY-1, phSE-5 and phage cocktail ELY-1/phSE-5 during 12 h.

The results obtained by the OD measurements were in concordance with those obtained by the colony counts for all cases (Figure 5A–C). The rate of bacterial growth of the two pure cultures and of the mixed culture of the two bacteria in the presence of single phage suspensions and of the cocktail was significantly different (ANOVA, *p* < 0.05) from that obtained with the sensitive and resistant bacteria in the absence of the phages and resistant bacteria with phages.

## 4. Discussion

Even though the major advantage of phage treatment is the phage specificity, since the non-target bacterial populations should remain undisturbed, phages should be capable to lyse the majority of strains of a given bacterial species [64]. Moreover, phages with a broader host range, that affect pathogenic bacteria of other species or even of other genera, can also be useful when the involved bacterium is not yet identified. Consequently, before using phages to treat infections caused by Enterobacteriaceae, like *E. coli* and *S*. Typhimurium, which are by far most commonly isolated microorganism in the clinical laboratory, the *in vitro* dynamics of phage-host replication characterization is important to decide if phage cocktails including strains specific to these bacteria can be effective to control infections caused by *E. coli* or *S*. Typhimurium. 

The results of this study indicate that both phages, phSE-5 (produced on *Salmonella*) and ELY-1 (produced on *E. coli*), can control bacterial strains of *E. coli* and *Salmonella*, two closely related genera of the Enterobacteriaceae family. However, none of the two phages infected other bacterial strains of the Enterobacteriaceae genera, nor other strains belonging to other bacterial families. Phages ELY-1 and phSE-5 formed completely cleared zones (spot test results) on 9 and 25 of the 50 tested strains, respectively. However, the efficiency of plating (EOP) results indicate that phages ELY-1 and phSE-5, besides their hosts, only formed lysis plaques on 4 strains of the tested bacterial strains. Phage ELY-1, besides its host, also infected 3 *E. coli* strains (*E. coli* ATCC 25922, *E. coli* Scc 34, and *E. coli* Scc69) and 1 *Salmonella* strain (*S.* Typhimurium ATCC 13311). Phage phSE-5, besides its host, infect also four non-*Salmonella* strains (bioluminescent *E. coli*, *E. coli* AE11, *E. coli* Scc 34, and *E. coli* Scc 69), which belong to different bacteria genera, but belong to the family of the Enterobacteriaceae. Mirzaei and Nilsson (2015) obtained analogous results and stated that the selection of phages cannot be performed by spot test and should be replaced by the EOP assays [65]. Negative EOP and positive spot test outcomes can occur when an overload of phages simultaneously infects a bacterium, leading to lysis due to the presence of high concentrations of lysins (“lysis from without”) [66], avoiding the replication of the phages by the bacteria [67,68,69,70]. Phages ELY-1 and phSE-5 respect the specificity criterion, so important in phage therapy, however, both phages have quite narrow host ranges. In the future, new phages need to be isolated and tested together with phages ELY-1 and phSE-5 to produce a cocktail with a broader spectrum of activity towards *E. coli* and *S.* Typhimurium

Although ELY-1 and phSE-5 showed low efficiency of plating against *S*. Typhimurium and *E. coli*, respectively, the obtained rate of inactivation was high. The maximum inactivation of phage ELY-1 was 4.5 log CFU/mL for *E. coli* and 2.2 log CFU/ml for *S.* Typhimurium. When phage phSE-5 was used to inactivate *S.* Typhimurium, after 4 h of incubation, the rate of inactivation was higher (2.2 log CFU/mL) than that observed against *E. coli*. However, the maximum inactivation of phage phSE-5 was higher against *E. coli* than against *Salmonella* (~3.8 log CFU/mL reduction for *E. coli* and 2.6 log CFU/mL for *S.* Typhimurium), but its effect on *E. coli* starts later (only after 8 h of treatment) than that of phage ELY-1 (inactivation starts after 2 h of treatment). 

The use of several phages in the form of cocktails increases their potential against pathogenic bacteria. Moreover, if more phages were included in the cocktail, the greater its potential would be for presumptive usage, i.e., prior to identification of the pathogens. However, as the number of phages in the cocktail increases, the possible impact on non-target bacteria is higher. Even so, like in most cases, this impact will be minimal when compared to what is expected from typical commercial antibiotics [49]. We also have to consider that too many phages per formulation can result in higher development and manufacturing costs and using multiple phages of the same genus with different host ranges in the cocktail may result in phage recombination and generate new host specificities [71]. 

The phage cocktail used in this study efficiently inactivated the two tested bacteria, either in mixtures or individually. The phage cocktail was more efficient to control *S.* Typhimurium and the mixture of bacteria than the single phage suspensions. Moreover, in both cases, the inactivation was observed sooner when the cocktail was used. When the phage cocktail was used to inactivate *S.* Typhimurium and the mixture of bacteria, after 2 h of treatment the rate of inactivation was higher (2 and 1.2 log CFU/mL, respectively) than the single phage suspensions (bacterial concentration similar to the bacterial control). These results are in accordance with other studies [50,72,73,74] that achieved a faster and higher inactivation by using phage cocktails over single phage suspensions. However, the phage cocktail was no more effective to inactivate the *E. coli* than the single suspension of phage ELY-1, but the efficiency of *E. coli* inactivation by the cocktail was similar to that obtained with the single phage suspension. As the purpose of using the cocktail resides in its application to inactivate both bacteria, that is, to be used prior to the identification of the pathogen involved in an infection, this is not a negative aspect. 

Phage therapy with phage cocktails, as well as with single-phage suspensions, did not prevent the bacterial regrowth after treatment. However, as stated before, for *S.* Typhimurium and for the mixture of bacteria, the use of the cocktail retarded the regrowth of bacteria, which is an important achievement. Moreover, the use of the phage cocktail limited the emergence of phage-resistant mutants in the mixture of the two bacteria. The development of resistant mutants in the mixture of bacteria against the phage cocktail ELY-1/phSE-5 (2.75 × 10^−4^) was lower than those obtained with phages ELY-1 (4.66 × 10^−4^) and phSE-5 (5.09 × 10^−4^). However, when the phage phSE-5 was used alone against its host as well as against *E. coli*, the emergence of resistance was higher than when the phage ELY-1 was used alone, reaching values of 10^−3^ CFU/mL. These results suggest that the presence of the phage phSE-5 in the phage cocktail increases the rate of resistant mutants against its host and *E. coli*. The frequency of emergence of phage resistant mutants using the phage ELY-1 against *E. coli* and against *S.* Typhimurium was significantly lower, 10^−5^ CFU/mL, a value similar to those observed before in other studies [32,75].

Some authors have suggested that phage exposure could cost bacteria their fitness, which can lead to their removal from the environment at a faster rate than their wild-type parents [76,77]. In this study, the experiment results of the fitness of phage resistant mutants showed that the concentration of sensitive bacteria and resistant mutants, when grown in the absence of phages ELY-1, phSE-5, and the phage cocktail ELY-1/phSE-5, are not different. However, these experiments were done in nutrient rich medium (culture medium) and without the presence of competition, from which, according to some authors, the cost of resistance can vary across environmental factors and degree of competition for resources [78,79]. Further studies are necessary to evaluates the cost of resistance *in vivo*.

In the near future, it will be essential to understand the efficiency of these single phage suspensions and cocktails *in vivo*, using, for instance, *G. mellonella* larvae as an animal model. According to several authors, *in vitro* experiments are not satisfactory to distinguish phage–bacteria interactions *in vivo* [38,44,80,81]. Numerous factors can affect the survival and lytic properties of the phages *in vivo*, e.g., bacteriophages/target bacteria ratio, the way and moment of treatment, physical and chemical properties, the neutralization of phages, and accessibility to target bacteria [82].

## 5. Conclusions

The use of a phage cocktail against the Enterobacteriaceae increases their potential to be used prior to the identification of the pathogenic bacterium, retarding even the regrowth of bacteria. However, the development of phage-resistant mutants is not prevented. Nevertheless, further studies are needed to understand the true potential of the use of these two phages and of the cocktail phages to control *E. coli* and *S.* Typhimurium, namely *in vivo* studies using animal models.

## Figures and Tables

**Table 1:** Host range of phages ELY-1 and phSE-5 determined on 50 Enterobacteriaceae strains. Clear lysis zone (+) and not lysis zone (−). EOP values are the mean of three measurements, followed by their standard deviation. The plating with the host strain was considered as EOP = 1.

Strains	ELY-1		phSE-5	
	Spot Test	EOP	Spot Test	EOP
*S.* Typhimurium ATCC13311	+	5.7 × 10^−4^ ± 2.5 × 10^−5^	+	1 (host)
Bioluminescent *E. coli*	+	1 (host)	+	2.2 × 10^−1^ ± 1.5 × 10^−2^
*Citrobacter freundii* 6F	−	0	+	0
*Enterobacter cloacae*	−	0	+	0
*E. coli* AC5	−	0	+	0
*E. coli* AD6	−	0	+	0
*E. coli* AE11	−	0	+	1.7 × 10^−8^ ± 5.6 × 10^−9^
*E. coli* AF15	−	0	−	0
*E. coli* AJ23	−	0	+	0
*E. coli* AN19	−	0	+	0
*E. coli* ATCC 13706	−	0	+	0
*E. coli* ATCC 25922	+	22.7 ± 1.02	+	0
*E. coli* BC30	+	0	+	0
*E. coli* BM62	−	0	+	0
*E. coli* BN65	−	0	+	0
*E. coli* Scc 09	−	0	−	0
*E. coli* Scc 33	−	0	−	0
*E. coli* Scc 34	+	5.5 × 10^−5^ ± 5.0 × 10^−6^	+	1.8 × 10^−5^ ± 1.5 × 10^−6^
*E. coli* Scc 35	−	0	−	0
*E. coli* Scc 36	−	0	−	0
*E. coli* Scc 37	+	0	−	0
*E. coli* Scc 38	−	0	−	0
*E. coli* Scc 40	−	0	−	0
*E. coli* Scc 41	−	0	−	0
*E. coli* Scc 43	−	0	−	0
*E. coli* Scc 45	−	0	−	0
*E. coli* Scc 47	−	0	−	0
*E. coli* Scc 48	−	0	−	0
*E. coli* Scc 49	−	0	−	0
*E. coli* Scc 50	−	0	−	0
*E. coli* Scc 51	+	0	−	0
*E. coli* Scc 52	−	0	−	0
*E. coli* Scc 53	−	0	−	0
*E. coli* Scc 55	−	0	−	0
*E. coli* Scc 56	−	0	−	0
*E. coli* Scc 58	−	0	−	0
*E. coli* Scc 69	+	2.8 × 10^−1^ ± 8.7 × 10^−2^	+	2.5 × 10^−8^ ± 4.8 × 10^−9^
*E. coli* Scc 77	−	0	−	0
*E. coli* Scc 78	−	0	−	0
*E. coli* Scc 91	−	0	−	0
*Proteus mirabilis*	−	0	+	0
*Providencia* sp.	−	0	+	0
*S.* Enteriditis CVA	−	0	+	0
*S.* Enteriditis CVB	−	0	+	0
*S.* Enteriditis CVC	−	0	+	0
*S.* Enteriditis CVD	+	0	+	0
*S.* Enteriditis CVE	−	0	+	0
*S.* Typhimurium ATCC 14028	−	0	+	0
*S.* Typhimurium WG49	−	0	+	0
*Shigella flexneri* DSM 4782	−	0	−	0

**Table 2:** Frequency of bacteria spontaneous phage-resistant mutants.

Bacteria	Phage	Frequency of Phage-Mutants
*E. coli*	phSE-5	1.29 × 10^−3^ ± 3.15 × 10^−5^
	ELY-1	5.06 × 10^−5^ ± 6.55 × 10^−6^
	ELY-1/phSE-5	1.22 × 10^−3^ ± 6.53 × 10^−5^
*S.* Typhimurium	phSE-5	2.80 × 10^−4^ ± 3.44 × 10^−5^
	ELY-1	5.11 × 10^−5^ ± 6.60 × 10^−6^
	ELY-1/phSE-5	2.75 × 10^−4^ ± 4.10 × 10^−5^
Mixture of *E. coli*/*S.* Typhimurium	phSE-5	5.09 × 10^−4^ ± 6.99 × 10^−5^
	ELY-1	4.66 × 10^−4^ ± 8.44 × 10^−5^
	ELY-1/phSE-5	6.45 × 10^−5^ ± 1.11 × 10^−5^
